# Isotopic Tracer for Absolute Quantification of Metabolites of the Pentose Phosphate Pathway in Bacteria

**DOI:** 10.3390/metabo12111085

**Published:** 2022-11-09

**Authors:** Khairunnisa Mohd Kamal, Mohd Hafidz Mahamad Maifiah, Yan Zhu, Nusaibah Abdul Rahim, Yumi Zuhanis Has-Yun Hashim, Muhamad Shirwan Abdullah Sani

**Affiliations:** 1 International Institute for Halal Research and Training (INHART), International Islamic University Malaysia (IIUM), Jalan Gombak 53100, Selangor, Malaysia; 2 Infection and Immunity Program, Department of Microbiology, Biomedicine Discovery Institute, Monash University, Victoria 3800, Australia; 3 Faculty of Pharmacy, University of Malaya, Kuala Lumpur 50603, Selangor, Malaysia

**Keywords:** metabolomic, bacterial metabolomics, pentose phosphate pathway (PPP), isotope labeling, absolute quantification

## Abstract

The pentose phosphate pathway (PPP) plays a key role in many metabolic functions, including the generation of NADPH, biosynthesis of nucleotides, and carbon homeostasis. In particular, the intermediates of PPP have been found to be significantly perturbed in bacterial metabolomic studies. Nonetheless, detailed analysis to gain mechanistic information of PPP metabolism remains limited as most studies are unable to report on the absolute levels of the metabolites. Absolute quantification of metabolites is a prerequisite to study the details of fluxes and its regulations. Isotope tracer or labeling studies are conducted in vivo and in vitro and have significantly improved the analysis and understanding of PPP. Due to the laborious procedure and limitations in the in vivo method, an in vitro approach known as Group Specific Internal Standard Technology (GSIST) has been successfully developed to measure the absolute levels of central carbon metabolism, including PPP. The technique adopts derivatization of an experimental sample and a corresponding internal standard with isotope-coded reagents to provide better precision for accurate identification and absolute quantification. In this review, we highlight bacterial studies that employed isotopic tracers as the tagging agents used for the absolute quantification analysis of PPP metabolites.

## 1. Introduction

The pentose phosphate pathway (PPP) is crucial in many cellular metabolic functions. The pathway is important for amino acid and nucleotide biosynthesis, metabolic processes regulation, oxidative stress and redox homeostasis prevention, and stress response activation [1,2,3]. PPP is required for regulating energy metabolism by producing nicotinamide adenine dinucleotide phosphate (NADPH), adapting metabolic reconfiguration in the cell cycle, generating response signaling pathways in cancer metabolism, and in host-pathogen interactions of parasitic protozoa and bacterial infections [1,2,4]. Studies also discovered that the metabolites of PPP are responsible for the regulation of human clear cell-renal cell carcinoma (ccRcc) redox homeostasis [5], type 2 diabetes mellitus [6], and as biomarkers of pancreatic cancer [7]. Recent findings reported significant perturbations of PPP metabolism in pathogenic bacteria upon antibiotic treatment [8,9,10,11].

PPP metabolism has been well studied over the years using various methods of analysis to gain an in-depth understanding of its function and regulation. Nonetheless, detailed understanding of the mechanistic information of PPP remains the interest of much research as most studies are unable to record the absolute quantification analysis of its intermediate metabolites. The absolute concentration levels of metabolites of PPP immediately reflect the actual metabolic conditions of a cellular system. A number of quantification methods have been used to measure the levels of targeted metabolites, but each comes with some limitations. The major challenges are due to the rapid turnover rates of the intermediates, with low abundances and poor separation between the sugar phosphates during analysis [2]. The developed method based on the incorporation of isotopic tracers as tagging agents coupled with high-tech mass spectrometry (MS) has significantly improved the accuracy of quantitative analysis [12,13,14,15].

Isotope tracer or labeling is not an entirely new approach used in metabolite identification and quantification as well as in flux analysis [16]. Metabolite labeling refers to a method used for incorporating detection or affinity tags into biomolecules that can be performed via in vivo endogenous synthesis and in vitro [17]. The incorporation of isotopic tracer techniques coupled with advanced analytical instruments has offered great advantages to both untargeted and targeted metabolomic approaches [18]. The technique is robust, which provides better separation between the identical compounds of interest. It allows accurate identification and direct quantification of metabolites’ levels and determination of pathway fluxes [19,20], leading to novel biomarker discoveries and metabolic pathway mapping [18]. In this review, absolute quantification of PPP metabolites, notably in bacteria studies, is discussed using isotopic tracer methods. The scope of this review covers sample preparation procedures in vivo and in vitro isotopic labeling studies, but it does not go into detail about the analytical equipment used.

## 2. Pentose Phosphate Pathway

The metabolic reactions in PPP are divided into oxidative and non-oxidative phases, where the activities majorly occur in the latter phase [21]. The oxidative phase is essential for the conversion of glucose 6-phosphate into carbon dioxide, ribulose 5-phosphate, and NADPH, which explains the vastly active reactions in the majority of eukaryotes [2]. The non-oxidative phase plays roles in the ageing process, regulating redox balance during stress, yielding ribose 5-phosphate for synthesis of nucleic acids and amino acid precursors, and glycolysis [2]. Other significant metabolites in the non-oxidative phase are erythrose 4-phosphate and sedoheptulose 7-phosphate. The former is important for aromatic amino acids synthesis and vitamin B6 metabolism [2,4,22,23] while the latter connects the intermediates of the glycolysis pathway and the non-oxidative PPP phase [2]. An alternative L-type PPP involves other intermediates, including octulose 8-phosphate, but little is known about its details [24]. A number of studies have shown that PPP is significantly involved in many important metabolic processes, including in cancer [25], cell proliferation [26,27], brain energy metabolism [28], host-pathogen response [29], targeted pathway of drug treatment against parasitic and bacterial infection [30,31], biosynthesis of bacteria’s lipopolysaccharides [32], and central pathway for bacterial infection [33].

Many recent bacterial metabolomics studies revealed that the levels of PPP metabolites were significantly changed by antibiotic treatments, including gluconate 6-phosphate, D-ribose 5-phosphate, D-erythrose 4-phosphate, glyceraldehyde 3-phosphate, D-sedoheptulose 7-phosphate, D-glucono-1,5-lactone 6-phosphate, and D-fructose 1,6-biphosphate [8,9,10,34,35,36,37]. For example, global profiling of *Acinetobacter baumannii* and *Pseudomonas aeruginosa* treated with combinations of antibiotics demonstrated significantly reduced levels of D-ribose 5-phosphate, D-erythrose 4-phosphate, and D-sedoheptulose 7-phosphate over 1 h of treatment [8,9,10,34,38]. The findings are essential and a detailed understanding of the metabolic changes is further required as this potentially provides a novel strategy of metabolite-based treatment in addressing the issue of antimicrobial resistance. Though the detailed analysis is warranted, our literature search indicates that only a few publications, particularly of bacterial studies, reported the results in the form of absolute concentrations of PPP metabolites (Table 1).

## 3. Isotopic Tracer

Detailed pathway analysis and development of the kinetic model of a particular metabolic pathway requires an accurate and reliable method to quantify the absolute levels of metabolites involved. Various analysis methods used for metabolite quantification have been reported, including measuring released radioactive carbon dioxide [43], colorimetric assays [44,45], combination of thin layer chromatography with ^14^C-labeled substrates [46], fluorometric method by enzymatic interconversions [44,47], and feeding with isotope-labeled glucose [2]. Nonetheless, these methods have some limitations in that they are time consuming, complex, have low sensitivity and applicability, are not robust, and are ineffective for distinguishing between analytes in the oxidative and non-oxidative phases [2].

**Figure 1:**
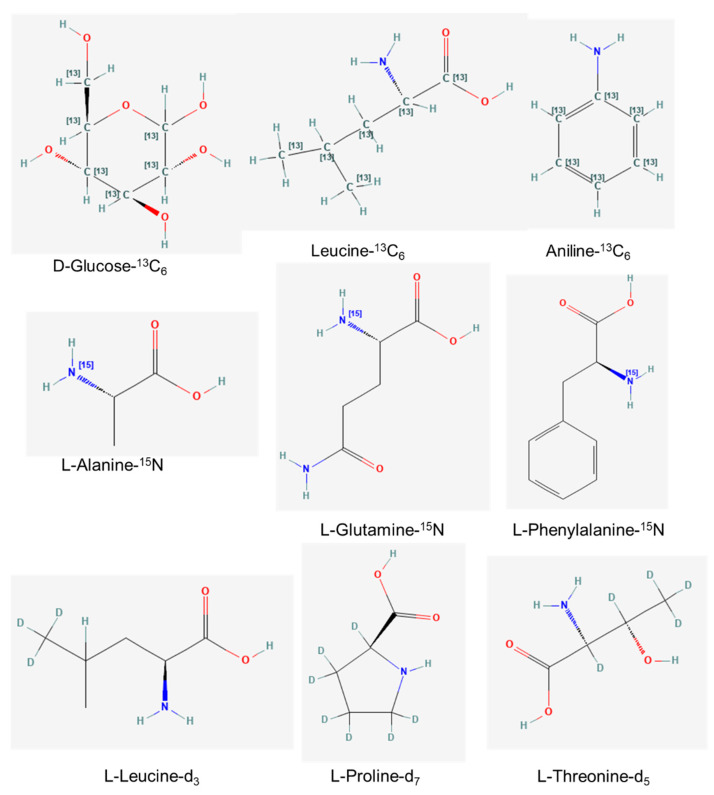
Examples of commercial stable isotope tracers of carbon (^13^C), nitrogen (^15^N), and deuterium (^2^H).

Isotopic labeling or tracer has been routinely used to identify and quantify the absolute levels of metabolites of interest [48]. Isotope-labeled compounds can be traced through the cellular metabolic pathways and reactions using appropriate analytical tools, including NMR and advanced MS methods. Its applications are essential for determining metabolic fluxes, generating kinetic and metabolite network modeling, and elucidating cellular mechanisms of action [49,50,51,52]. Stable, non-radioactive isotopes are versatile as they are naturally occurring and stable over a period of time without spontaneous decay and emission of radiation [53]. Available isotope tracers include carbon labeled (^13^C), deuterium (^2^H), and nitrogen (^15^N) (Figure 1). Carbon labeled is the most versatile as deuterium may decrease labeling grade during storage due to exchange of the deuterium molecule in hydrogen labile (H^+^) solvent [54]. Moreover, deuterium labeling on fatty acids may be lost during the desaturation phase and is unsuitable for in vivo studies [54,55]. It is also reported that the use of deuterium isotope limits the application with LC-MS due to the co-elution effects which affect the chromatographic outcomes [56]. As for nitrogen labeled, the usage is limited to nitrogen-containing molecules [54].

Isotope compounds are chemically identical but different in mass, the mass difference between the heavy isotope tracer and the naturally occurring molecule can be detected and quantified [53]. The isotopic labeling method has been used to generate an Internal Standard (IS) for compounds of interest to increase metabolite accessibility [13,14]. This has been recognized as a gold standard to produce reference standards for accurate identification and quantification of targeted metabolites [18]. This helps to reduce common issues during MS analysis, such as ionizing inefficiency and inaccuracy in metabolite quantification caused by sample co-elute matrices [15].

The isotope-labeled metabolite sample, together with a known concentration of isotope-labeled IS, enables the quantification of the absolute concentration of the desired compounds [15,21]. Direct quantitation of the metabolite of interest is achievable by the ratio comparison of the chromatogram peak intensities formed between the labeled-metabolite and IS with known concentration [21,41]. A number of tagging agents, including glucose and aniline, have been used to target different functional groups in metabolites with better selectivity while allowing universal detection of other metabolites [15].

Previous studies reported the prime isotope analog of U-^13^C labeled was commonly used as IS in detecting PPP metabolites, as summarized in Table 1. Chemical derivatization is proven to be effective in overcoming the common issue of poor separation of complex biological samples in chromatography analysis [12]. Tagging the metabolite with isotopes increased the hydrophobicity of the compound, thus, improving the detection and separation outcomes of liquid chromatography-mass spectrometry (LC-MS) [57]. The presence of hydrophobic elements retained polar metabolites in the column, resulting in better separation, peaks formation, and retention time [57]. The compounds or metabolites of the experimental sample are labeled with a different isotope of an identical tag (i.e., ^13^C-glucose, ^13^C-glutamine, and 1-^2^H or 3-^2^H-glucose) before the reaction is halted [13,15,48].

A step of chemical derivatization in the labeling process minimizes problems during MS analysis including in achieving the same ionization energy between standard and sample, inaccuracy in metabolite quantification due to its diversity in functional groups, and co-eluting matrix of the sample [15]. The ionization efficiency can be enhanced by adjusting the physical properties of tagged functional groups of metabolites, thus increasing the separation resolution as well as the sensitivity for LC-MS analysis [15]. Derivatization with isotope labeling also improves the detectability of low abundance molecules by increasing the mass of the molecule, therefore avoiding possible interference with the background molecules and impurities present at low *m/z* area [12]. In addition, compared to the conventional method of isotope synthesis of individual metabolites, isotope tagging allows simultaneous analysis of multiple samples in a single run and overcomes the limitations in obtaining commercial stable isotopes [15].

### 3.1. Studies of PPP Metabolism by Isotopic Tracer Method

The application of the isotope labeling technique enabled the study of metabolic fluxes and regulatory pathways of PPP in different types of cells [23,58,59,60,61,62,63,64]. ^13^C-glucose as the isotopic tracer is most commonly found in literature due to its versatility in tagging of metabolites [65]. Lee et al. (1998) used [1,2-^13^C_2_]-glucose to determine PPP metabolism and estimate the relative metabolite fluxes through enzymatic reactions of transaldolase and transketolase in human hepatoma cells. [1,2-^13^C]-glucose isotopomer was also employed to re-examine the central metabolic pathways of *Clostridium acetobutylicum* [60]. A study demonstrated that [2,3,4,5,6-^13^C]-glucose was the most suitable substrate in interpreting oxidative PPP metabolite fluxes in mammalian cells of HEK-293 cells compared to [1-^13^C]-glucose [61]. Clasquin et al. (2011) highlighted the use of 6-^13^C-glucose to elucidate the alternative mechanism of riboneogenesis in yeast to balance the needs of redox biosynthesis and homeostasis [23]. A study by Wushensky et al. (2018) successfully elucidated pathways involving PPP metabolism of *B. megaterium* by adding the samples into U-^13^C_6_-glucose agar plates.

Previous studies reported the use of deuterium-labeled glucose of 1-^2^H or 3-^2^H-glucose to detect the utilization of oxidative PPP metabolites in producing NADPH [62,66]. Deuterium as a tagging agent was able to overcome the limitations of carbon isotopes in allowing direct quantification of NADPH redox activity [62,66]. Furthermore, isotopic labeling has also aided in discovering unknown cellular activity in central carbon metabolism [58,64,67]. For instance, the use of 1-^13^C-xylose was successful in revealing the existence of alternative mechanisms in *E. coli* that are activated by the buildup of sedoheptu-lose-7-phosphate [64]. The purpose of the alternative process was highlighted to maintain the demands of the primary carbon metabolism fluxes [64]. In another study, labeling the Chinese hamster ovary (CHO) cells with [1,2-^13^C] glucose detected the upregulation of fluxes of oxidative PPP to drive transketolase like reaction and reduce oxidative stress during the stationary phase [58,67].

### 3.2. In Vivo Synthesis of Metabolite-Labeled Isotopes

In vivo isotope labeling is accomplished by culturing the cells or organisms in conditions containing a tagged chemical analogue of a certain natural molecular building block (e.g., amino acid, nucleotide, and carbohydrate) [68]. The presence of the chemical analog is used to label the metabolic networks and its related metabolites [57]. Stable isotope labeling is a non-radioactive approach used to detect small changes by measuring the differences in relative metabolites levels between light and heavy labeling [69,70]. Other common approaches of in vivo metabolic labeling are through radioactive labeling [71], photoreactive amino acids [72], and fluorescent probes in live cells [73]. Radioactive metabolic labeling is easy to detect and affordable, but there are concerns about safety hazards, generated waste, and toxicity [71]. Photoreactive amino acids create in vivo crosslinking via covalent bonds with any amino acid side chain or peptide bone. It does, however, have lower integration rates than isotope analogs and less tolerance over long time periods [72,74]. The use of fluorescent probes in live cells enables robust detection of signals with high sensitivity and versatility, yet requires specialized instruments for the detection of labeled metabolites [73].

Commercial isotope analogs of analytes are very limited and can be very costly, especially for metabolites of the central carbon metabolism [13,14]. In addition, the process of in vivo synthesis of the desired isotope labeling is laborious as it requires feeding the culture with U-^13^C-labeled substrate for a period of time and correction steps for any incomplete labeling [13,14,39,40]. The microorganism under the study is cultivated in two different mediums, with and without the ^13^C-labeled medium such as U-^13^C-glucose. Culture fed with the ^13^C-labeled medium serves as the IS. Rapid mixing of both cultures is then performed and proceeds for further analysis. Mashego et al. (2004) previously introduced a method known as Mass Isotopomer Ratio Analysis of U-^13^C-Labeled Extracts (MIRACLE). Known amounts of U-^13^C-labeled cells are introduced to unlabeled cell samples prior to extraction. The enrichment of heavy tagged isotopic metabolites may serve as the IS for all intracellular metabolites to be quantified [14,57]. Metabolites are quantified through peak area ratios comparison of the ^12^C- and U-^13^C-labeled metabolites samples and IS-based calibration lines [13,14]. The common ion suppression events in LC-ESI-MS/MS have no influence on the coelution peak area of the U-^12^C-labeled molecule to its U-^13^C-labeled equivalent IS in the LC [14]. Therefore, the non-linear response of electrospray ionization of MS is eliminated by the coextraction steps [14].

Incomplete tagging of metabolites has been an issue in isotopes labeling. The problem commonly occurs in a conventional labeling method when culturing the organism in U-^13^C-labeled medium [14]. Although incomplete or unlabeled metabolites still can be detected during analysis, the result may affect the accuracy and compromise the data. The certainty of metabolites tagging cannot be measured as some metabolites may be detected in low intensity while some structurally similar metabolites could not be differentiated by MS [41].

### 3.3. In Vitro Synthesis of Metabolite-Labeled Isotopes

The synthesis and labeling of metabolites with stable isotopes are possible through an in vitro process. Yang et al. (2008) developed a method known as a Group Specific Internal Standard Technology (GSIST) which applied isotopic aniline for labeling of metabolites of central carbon and energy metabolism including the PPP. Compared to common in vivo synthesis, this method is simple and able to target multiple functional groups of metabolites. Aniline as a chemical derivative tag can be used for both relative quantification of unknown compounds and absolute quantification of known compounds without requiring the addition of cultured ^13^C-coded internal standards prior to analysis [41]. The derivatization of isotope labeling is required and takes place at the aldehyde and phosphate groups of the sugar phosphates. Two isotope labeling agents of chemically identical but different stable isotope compositions are used such as ^12^C-labeled for the sample (or metabolite reference standard) while ^13^C-labeled for the IS.

Tagging the samples with isotope agents allows sample components to be chemically coded according to their origin [41]. The derivatization of the metabolites allows each molecule in the samples to serve as IS which helps in determining the concentration of compounds of interest in the experiment by ratio comparison of peak intensity [41]. Co-elution of identical isotope labeling agents (i.e., ^12^C-labeled samples and ^13^C-labeled IS) at the same retention times (RT) results in a pair of peaks in MS. This condition aids in distinguishing the interfering matrix, such as metabolite peaks from background signals, noise, or unlabeled metabolite signals, resulting in improved confidence and selectivity of extraction pair information [12]. The use of ^12^C-aniline and ^13^C-aniline as the tagging agents has been successfully used to identify and quantify metabolites of interest including those from PPP metabolism [21,42]. Jannasch et al. (2011) applied the similar protocol in quantifying PPP metabolites and intermediates of *S. cerevisiae*. Similarly, Vilkhovoy et al. (2019) successfully quantified cell-free protein synthesis (CFPS) of *E. coli*. The methodology adapted from the GSIST approach was capable of detecting and quantifying 40 compounds involved in the tricarboxylic acid cycle, PPP, energy metabolism, and cofactor regeneration in CFPS processes.

### 3.4. Aniline Tagging Method

**Figure 2:**
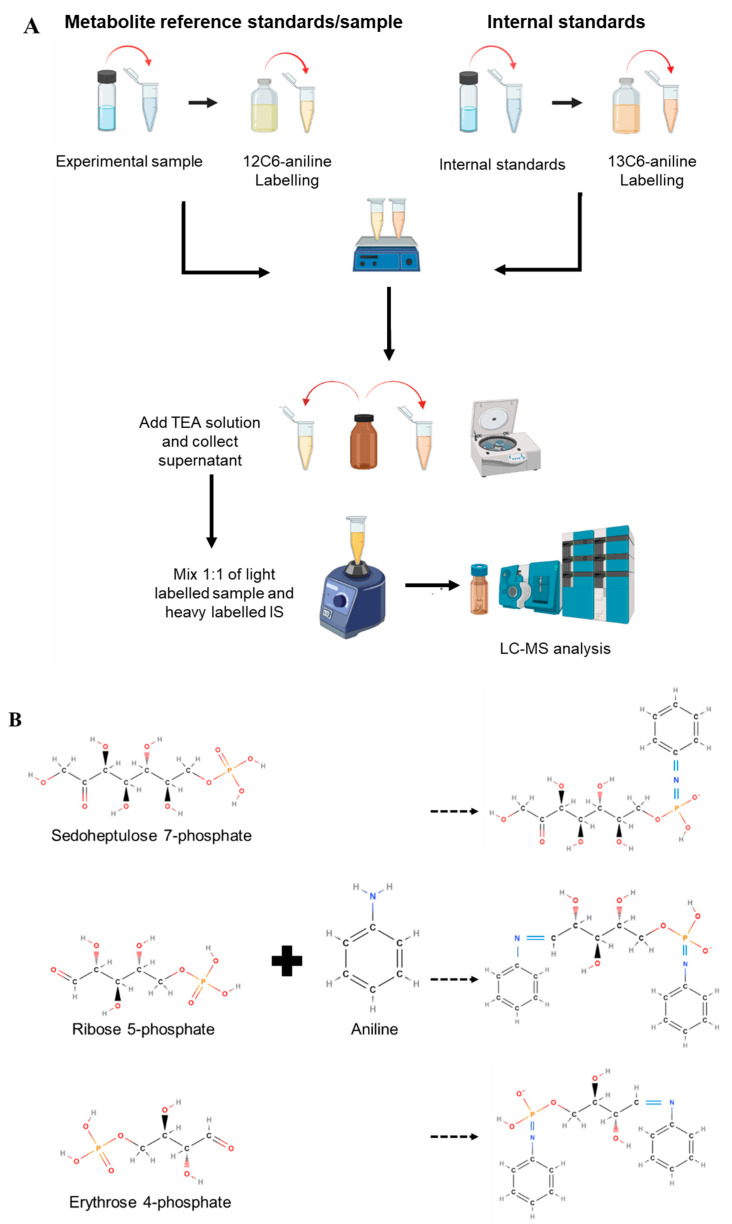
Strategy of aniline tagging method. (**A**) Preparation steps for stable isotope labeling of aniline for experimental samples and internal standards. (**B**) Tagging of PPP metabolites with aniline isotope. For R5P and E4P, one mole of aniline is attached to the phosphate and aldose groups, respectively. For S7P, only one mole of aniline is attached to the phosphate group.

Aniline is an aromatic amine with a colorless to slightly yellow liquid, rich in benzene derivative, and consists of a phenyl group attached to an amino group. Upon exposure to light or air, aniline may turn from a darker color of brown to red due to oxidation. The light and heavy labeling reagents of ^12^C_6_-aniline and ^13^C_6_-aniline, respectively, are prepared prior to the tagging procedure (Figure 2) [21]. Initially, an individual metabolite or compound (i.e., a commercially available metabolite reference standard) under a study is tagged with ^12^C_6_-aniline and its RT is determined to be set as a reference for the experimental sample. The IS is prepared by mixing the metabolite reference standard with the heavy label ^13^C_6_-aniline. To validate the aniline labeling reaction, the individual metabolite reference standard is labeled with aniline and the labeling pattern is observed by the spectrum in MS [41]. Experimental metabolite samples are tagged with ^12^C-aniline (light-labeled) and then mixed at equal ratios with the labeled-IS and further analyzed by MS. The metabolites of the sample are identified and quantified based on the peaks detected by the MS and then compared by ratio differences between the sample and the labeled IS. The samples and IS are expected to co-elute at the same RT, thus the mass difference between the two isotopes allows the distinction between the sample and IS area in the MS.

The absolute levels of metabolites are obtained by comparing the ratio between the intensity of the corresponding sample tagged with ^12^C_6_-aniline and IS tagged with ^13^C_6_-aniline peaks of the chromatograms. The results by Yang et al. (2008) indicated that the method successfully determined most of the intermediates involved in central carbon and energy metabolism of *S. cerevisiae* in a single run of reversed-phase LC-MS with high accuracy (RSD < 10%). In a separate study, using the same approach, structurally paired isomers of glucose-6-phosphate and fructose-6-phosphate were successfully separated in a single run of LC-MS [42]. The technique is practically used to quantify cell-free metabolism as well as whole-cell extracts [15,42]. The approach allows for the accurate identification and quantification of metabolites as the co-elution between the IS and experimental sample compounds and is able to eliminate the ion-suppression effects during LC-MS analysis [21,42]. Vilkhovoy et al. (2019) indicated that the GSIST procedure is only suitable for quantifying metabolites present in central carbon and energy metabolism, rendering it unsuitable for other essential pathways, such as amino acid and fatty acid metabolism. The method can be applied for the identification and absolute quantification of a diverse group of metabolites, including sugars, phosphosugars, carboxylic acids, nucleotides, and coenzymes.

## 4. Conclusions

Absolute quantification of metabolite concentration levels provides a detailed understanding towards the characteristics of metabolites as well as pathway changes under different conditions. As the method of absolute quantification has become a major challenge especially in bacterial metabolomics studies, isotope labeling coupled with MS has significantly improved the analysis. The use of a stable isotope tracer has facilitated the identification and absolute quantification of biomolecules and enhanced our understanding of metabolic fluxes. Importantly, the approach of the isotope tracer highlights the opportunity to elucidate novel cellular pathways of biological systems, such as antibiotic mechanisms of action, host–pathogen interaction, and biomarker discovery. Compared to the conventional in vivo method, an in vitro synthesis of isotope labeled-metabolites via GSIST introduced by Yang et al. (2008) allows for absolute quantification of metabolites in a more simple and robust method. The approach could serve as an alternative, especially in targeted metabolomics studies, with increased accuracy of metabolite quantification.

## Figures and Tables

**Table 1:** Absolute quantification of pentose phosphate pathway (PPP) metabolites of microbial samples using the isotope labeling method.

Isotopic Tracer	Sample	Method	PPP Metabolites	Reference
U-^13^C labeled medium	*Saccharomyces cerevisiae*	Rapid sampling and mix of chemostat labeled with ^12^C-labeled steady state glucose and ^13^C-labeled.	G6P, F6P	[14]
U-^13^C_6_-glucose	*S. cerevisiae*	Feed the culture with U-^13^C_6_-glucose and the sample is added to the unlabeled calibration standards as IS.	G6P, F6P	[13]
U-^12^C and U-^13^C-glucose	*Trypanosoma brucei*	Replacing the growth media with media containing ^12^C-labeled and ^13^C-labeled glucose.	R5P, F6P	[39]
U-^13^C_6_-glucose	*Bacillus megaterium*	Addition of sample into U-^13^C_6_-glucose agar plates, followed by the continuation of the culture at regular intervals for isotopic switches	G6P, F6P, 6PG, R5P, S7P, E4P	[40]
^12^C_6_- aniline and ^13^C_6_-aniline	*S. cerevisiae*	Tagging of internal standards with ^13^C_6_-aniline and derivatization of compounds in the sample with ^12^C^6^-aniline	G6P, F6P, DR5P, G3P, DE4P, DR5P	[41]
	*S. cerevisiae*		G6P, F6P, DR5P, G3P, 6PG, DE4P, DR5P, DS7P, X5P	[21]
	*Escherichia coli*		G6P, F6P, DR5P, G3P, 6PG, DE4P, DR5P, DS7P	[42]

G6P: Glucose 6-phosphate; F6P: Fructose 6-phosphate; R5P/DR5P: Ribose 5-phosphate/D-ribose 5-phosphate; 6PG: 6-phosphogluconate; S7P/DS7P: Sedoheptulose 7-phosphate/D-sedoheptulose-7-phosphate; E4P/DE4P: Erythrose 4-phosphate/D-erythrose 4-phosphate; X5P: Xylulose 5-phosphate; G3P: Glyceraldehyde 3-phosphate.
